# Imaging the node-linker coordination in the bulk and local structures of metal-organic frameworks

**DOI:** 10.1038/s41467-020-16531-y

**Published:** 2020-06-01

**Authors:** Boyuan Shen, Xiao Chen, Kui Shen, Hao Xiong, Fei Wei

**Affiliations:** 1 Beijing Key Laboratory of Green Chemical Reaction Engineering and Technology, Department of Chemical Engineering, Tsinghua University, Beijing, 100084 China; 2 School of Chemistry and Chemical Engineering, South China University of Technology, Guangzhou, 510640 China

**Keywords:** Metal-organic frameworks, Transmission electron microscopy

## Abstract

Porous metal-organic frameworks (MOFs) have shown wide applications in catalysis, gas storage and separation due to their highly tunable porosity, connectivity and local structures. However, the electron-beam sensitivity of MOFs makes it difficult to achieve the atomic imaging of their bulk and local structures under (scanning) transmission electron microscopy ((S)TEM) to study their structure-property relations. Here, we report the low-dose imaging of a beam-sensitive MOF, MIL-101, under a Cs-corrected STEM based on the integrated differential phase contrast (iDPC) technique. The images resolve the coordination of Cr nodes and organic linkers inside the frameworks with an information transfer of ~1.8Å. The local structures in MIL-101 are also revealed under iDPC-STEM, including the surfaces, interfaces and defects. These results provide an extensible method to image various beam-sensitive materials with ultrahigh resolution, and unravel the whole framework architectures for further defect and surface engineering of MOFs towards tailored functions.

## Abstract (web summary)

Metal-organic frameworks (MOFs) are so sensitive to electron beams that it is challenging to atomically image their structures by electron microscopy. Here, the authors use the integrated differential phase contrast technique in STEM to achieve the low-dose imaging of a beam-sensitive MOF, revealing its detailed local structure.

## Introduction

Metal-organic framework (MOF)^1–4^ is a typical class of porous materials consisting of diverse metal nodes and organic linkers. Due to their naturally tunable frameworks (porosity, connectivity, surfaces, and defects), MOFs have attracted attention in catalysis, gas separation, storage, and drug delivery^5–12^. Thus, understanding the relationship between the framework structures and functions of MOFs is one of the most important tasks for researchers in related disciplines. In reciprocal space, the diffraction methods using X-ray, neutrons, and electrons can provide periodic structural information with accurate positions of each atom in MOFs^13–15^. However, in real applications, we are mainly concerned about the aperiodic information (known as the local structures), including defects, interfaces, surfaces, and deformations. To investigate these local structures, real-space observations are highly demanded after a series of developments on imaging techniques. Among them, the scanning transmission electron microscope (STEM)^16–18^ is one of the most powerful imaging tools with the highest resolution of 50 pm, which has fully met the requirements to solve the current problems. However, the high-energy electrons will generate extrinsic defects in the specimens and even destroy the lattices of some beam-sensitive materials^19–23^. In addition, it limited the imaging of MOFs with high resolution^24–31^. Thus, for a long time in the past, we have always lacked sufficient understanding of the atomic local structures in very beam-sensitive MOFs.

How to reduce the influence of the electron beam while maintaining the current resolution and signal-to-noise ratio is an important issue for electron microscopy. Recently, some efficient progresses on the low-dose imaging technique were reported. For example, the direct-detection electron-counting camera was used to detect the low-dose electrons and achieve the real-space observation of MOFs in a high-resolution transmission electron microscope (HRTEM) with sub-unit-cell resolution^32–35^. Then, the cryogenic condition was further introduced to reduce the electron damage to the MOF crystals for a better imaging^36^. All these results required extra equipment beyond the available commercial STEM, which created barriers to the popularization of these techniques to match a wide range of application needs. In this work, we report a general STEM strategy to image the beam-sensitive MOFs with a high resolution and signal-to-noise ratio based on the two-dimensional (2D) integration of differential phase contrast (iDPC) images.

The iDPC technique is available in the new generation of Cs-STEM with DPC detector and has been reported to show a great potential for the low-dose imaging of beam-sensitive materials in highly crystalline structures^37–44^. Especially, the iDPC technique enabled the linear imaging of the projected electrostatic potential in lattice, which made the resulting contrast proportional to the atomic number and enhanced the contrasts of light elements comparable with the metal atoms^40^. In addition, the integration process of DPC-STEM image will naturally reject the non-integrable vector field, such as the noises, to obtain the corresponding iDPC-STEM image^39^. Thus, the signal-to-noise ratio of the iDPC-STEM images will be enhanced even under an ultralow electron-beam current. These advantages make the iDPC technique, probably the most efficient method for the atomic imaging of MOFs.

In this work, we successfully image the detailed structures of the MOF MIL-101 with an atomic resolution by iDPC-STEM. Based on these observations, we reveal the node-linker coordination in both the bulk and local structures of MIL-101. The surfaces and interfaces of MIL-101 crystals can especially be visually investigated from the imaging results. These results not only helped us to resolve the construction of MOF crystals but also provided a general method to image various beam-sensitive materials when we extended the iDPC-STEM imaging to other porous frameworks.

## Results

### The iDPC-STEM imaging of MIL-101 crystals

**Fig. 1:**
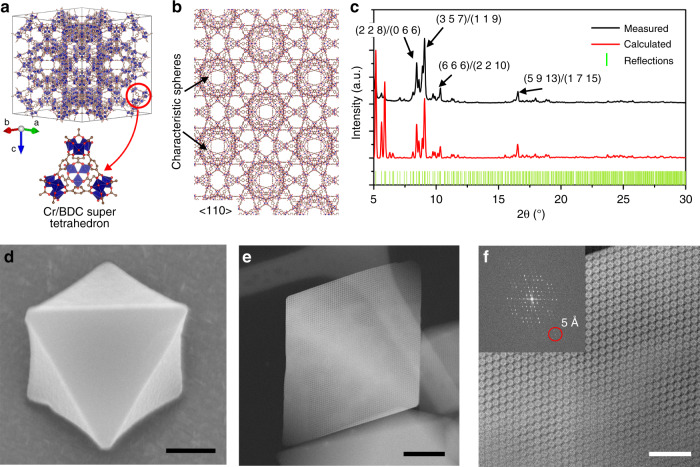
The octahedral MIL-101 nanocrystals. **a** The framework model of MIL-101. **b** The structural model of MIL-101 viewed from the <110> projection. **c** The PXRD results of MIL-101 crystals indicating a high crystallinity for the STEM imaging. **d** The SEM image showing the octahedral shape of a MIL-101 crystal. Scale bar, 200 nm. **e**, **f** The ADF-STEM images of MIL-101 from the <110> projection. The inset of **f** shows the corresponding FFT pattern with an information transfer of 5 Å. Scale bars, 100 nm in **e** and 30 nm in **f**.

The MOF specimen we used in this work for the iDPC-STEM imaging is MIL-101^45^, a typical stable crystalline MOF with the giant unit cells (89 Å) and cages. The MIL-101 framework is built by the corner-sharing super tetrahedrons consisting of the trimeric Cr nodes and 1,4-benzene dicarboxylate (BDC) linkers. Due to the large cage volumes and surface areas, the MIL-101 crystals were used as the supports for various metal atoms and particles in catalysis^46–48^. Thus, it is of great significance to atomically resolve the node-linker coordination in the local structures of MIL-101 for the further characterizations of these catalysts. Figure 1a, b and Supplementary Fig. 1 show the structural model of MIL-101 framework. Especially, the <110> projection exhibits the complicated characteristic spheres regularly arranged and marked in Fig. 1b, whereas the node-linker coordination between them can be individually resolved. These Cr/BDC super tetrahedrons between characteristic spheres helped revealing the connection of cages inside the frameworks, especially at the surfaces and interfaces.

First, we used the powder X-ray diffraction to confirm the periodic crystal structure and high crystallinity of MIL-101 (Fig. 1c). Furthermore, as we observed under the scanning electron microscope (SEM; shown in Fig. 1d and Supplementary Fig. 2), the as-prepared MIL-101 nanocrystals exhibit the regular octahedral shapes, which is benefit for searching the zone axes based on the projected shapes of crystals. For example, the octahedral crystals preferred to lie on the carbon film with their <110> zone axis parallel to the injected beam. Then, we used the annular dark field (ADF) STEM images (Fig. 1e, f and Supplementary Fig. 3) to show the morphology and lattices of MIL-101 crystals from the <110> projection. In Fig. 1f, the regularly arranged characteristic spheres of MIL-101 could be observed with the sub-unit-cell resolution. However, it is not enough to identify the node-linker coordination (Cr/BDC super tetrahedrons) between the spheres, as the inset fast Fourier transform (FFT) pattern shows an information transfer of only 5 Å. In addition, for the ADF-STEM imaging, we cannot further reduce the beam current (0.7 ~ 3.2 pA) due to the poor signal-to-noise ratio. Thus, the resolution breakthrough is quite difficult for this imaging technique.

**Fig. 2:**
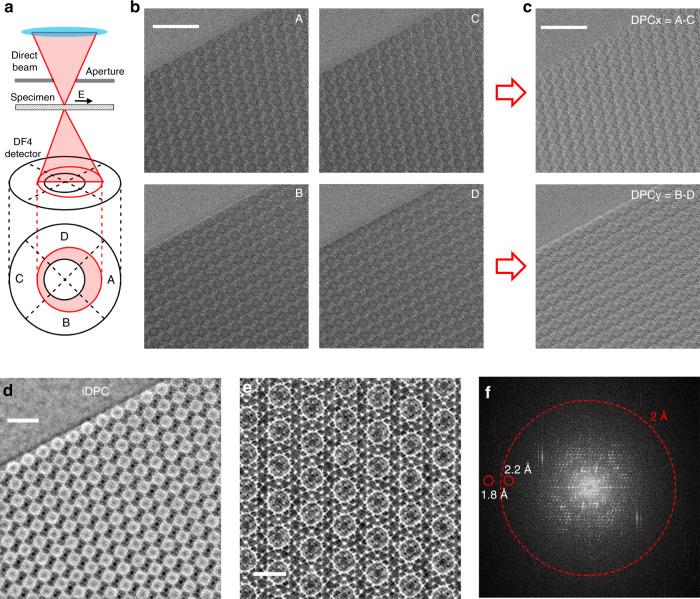
The iDPC-STEM imaging of MIL-101<110> projection with an ultrahigh resolution. **a** The schematic set**-**up of iDPC-STEM. The electron beam was deflected by the potential field in specimens and detected by the four segments of DPC detector. **b** Four images detected by the four segments (A–D) of DPC detector, respectively. Scale bar, 20 nm. **c** The DPC image obtained from the four images in **b**. Scale bar, 20 nm. **d** The iDPC-STEM image obtained by a 2D integration of the DPC image in **c**. Scale bar, 10 nm. **e** The magnified iDPC-STEM image with the highest resolution, which is perfectly matched with the structural model. Scale bar, 5 nm. **f** The corresponding FFT pattern of **e** in a log scale, showing an information transfer up to 1.8 Å.

**Fig. 3:**
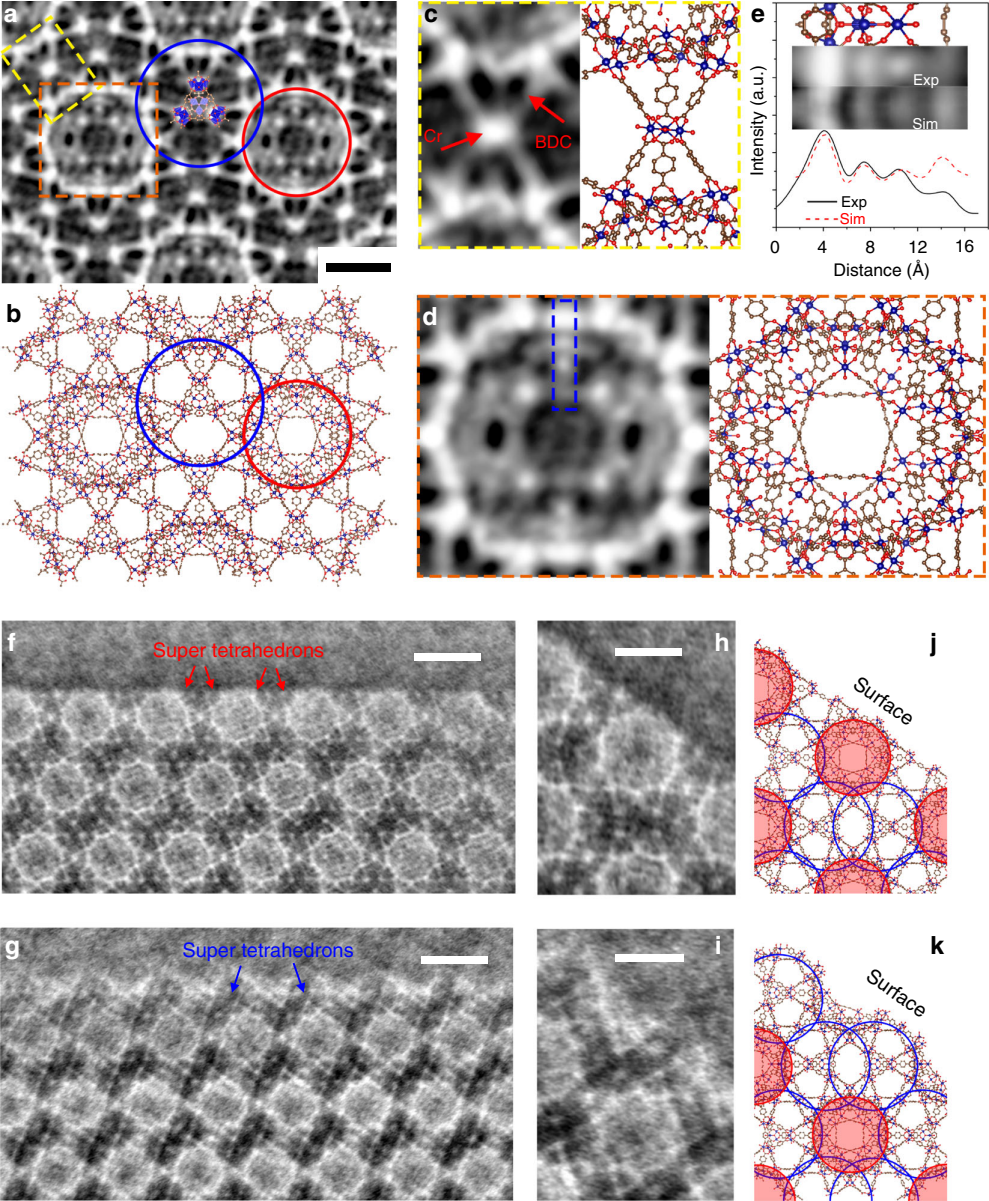
Imaging the atomic lattices and surface structures in the MIL-101 crystals. **a**, **b** Imaging the atomic MIL-101 lattice in the averaged iDPC-STEM image. The red and blue circles indicate two types of cages with 29 and 34 Å sizes, respectively. Scale bar, 1 nm. **c** Imaging the super tetrahedrons formed by Cr nodes and BDC linkers (marked as the yellow frame in **a**). **d** Imaging the structure in 29 Å cage in the MIL-101 (marked as the orange frame in **a**). **e** The profile analysis of image (exp) and simulation (sim) of the detailed structures in the blue frame in **d**, which helps to resolve the Cr columns in the frameworks. **f**, **g** Two types of surface terminations with two types of cages exposed on the {111} surfaces. Scale bars, 5 nm. **h**, **i** The structures of single-unit cells at two types of surface terminations, respectively. Scale bars, 3 nm. **j**, **k** The structural models of **h** and **i** showing the {111} surfaces terminated by different cages.

Then, we used the iDPC technique to pursue a higher resolution for MOF imaging. The set-up of iDPC-STEM is shown in Fig. 2a. The four figures (A–D) were obtained from the four segments of a commercial DPC detector, respectively (Fig. 2b). Then, in the situation shown in Fig. 2a, the *x* and *y* components of DPC images were calculated as DPC_*x*_ = *A* – *C* and DPC_*y*_ = *B* − *D* (Fig. 2c). In addition, in other non-canonical situations, the DPC_*x*_ and DPC_*y*_ components should be calculated more complexly considering the rotation angle between the scanning direction and the orthogonal direction of quadrants^39^. After a 2D integration, the obtained iDPC-STEM image could exhibit the detailed structures inside the MIL-101 framework (Fig. 2d). During the iDPC-STEM imaging, the used convergence semi-angle was 10 mrad and the operating voltage was 300 kV. Furthermore, the current of electron beam was reduced to <0.1 pA (corresponding to an electron dose of 40 *e*−/Å^2^), whereas the high signal-to-noise ratio can maintain due to the integration process. As shown in Fig. 2e, the magnified iDPC-STEM image provided much more details of the MIL-101 lattice, especially the clear Cr/BDC super tetrahedrons between the characteristic spheres, consistent with the structural model. In Fig. 3f, the corresponding FFT pattern obviously contains more reflection spots showing an information transfer of ~1.8 Å. This resolution has exceeded the previous results for MIL-101^24,27,35^, where the information transfer of 16 Å in ADF-STEM and 2.5 Å in HRTEM with the direct-detection electron-counting camera have been achieved. Thus, diverse imaging techniques have made great progress in recent years to improve the image resolution of MIL-101 by an order of magnitude from 16 Å to 2.5 Å then to 1.8 Å in this work, which provided more possibilities to further atomically resolve the local structures and light elements in MOFs.

Moreover, the averaged iDPC-STEM image in Fig. 3a showed an enhanced signal-to-noise ratio and provided the more detailed structures of super tetrahedrons with proper contrasts. The averaged image was obtained by overlapping the images periodically selected from the original image of Fig. 2e using the Smart Align software (Supplementary Fig. 4). This averaged image is perfectly matched with the structural model in Fig. 3b. The red and blue circles outlined two types of giant cages with the sizes of 29 and 34 Å, respectively, in the MIL-101 framework. These giant cages with smaller windows (12 and 16 Å) are significant to load the guest molecules and metal atoms for the gas separation, storage, and catalysis. The characteristic spheres in these images directly indicated the 29 Å cages. Furthermore, comparing the image and model in Fig. 3c (the area marked by the yellow frame in Fig. 3a), we can observe three BDCs in the single super tetrahedron owing to the high-contrast light elements imaged by the iDPC-STEM. In Fig. 3d (the area marked by the orange frame in Fig. 3a), the atomic structures inside the 29 Å cages can also be resolved, which is consistent with the models and simulations (Supplementary Fig. 5). Especially, in the profile analysis in Fig. 3e, the Cr atom columns in the area marked by the blue frame in Fig. 3d were identified by the peaks in the intensity profile. These results confirmed that the iDPC-STEM could help us to observe not only the Cr nodes with the atomic resolution but also the organic linkers in MOFs, which is of great significance to study the local structures, metal loading, adsorption, and catalysis in the MOFs in the real space.

### Resolving the surface structures of MIL-101

After observing various crystal surfaces (Supplementary Fig. 6) in details, we found two completely different types of surface terminations co-existing in the MIL-101 crystals (Fig. 3f, g). One of them exhibited the complete characteristic spheres along the {111} surfaces (Fig. 3f), whereas another terminated by near half of the spheres (Fig. 3g). We did not observe the surface organic ligands in these images. Very similar results have also been observed recently under the same synthesis conditions by the HRTEM^35^. Figure 3h, i show the detailed structures of single-unit cells at two types of surface terminations. To identify the surface structures by the iDPC-STEM images, we established five possible {111} surface models by reducing the nodes and linkers layer by layer from the [111] direction, and then obtained the simulated potentials without considering the status of surface organic ligands in Supplementary Fig. 7. After comparing the surfaces in Fig. 3h, i with these simulated potentials, we confirm that the models shown in Fig. 3j, k are most consistent with our observations, which may provide a better insight into these two different terminations. Two imaged {111} surfaces in Fig. 3h, i were terminated by different types of giant cages. That is, the complete 29 and 34 Å cages, marked by the red and blue circles, are exposed on the {111} surfaces in Fig. 3k, l, respectively. In Supplementary Fig. 8 and Supplementary Discussion, we calculated the energy of different surface models based on the numbers of surface dangling bonds. The theoretical results support our observations that the {111} surfaces of MIL-101 prefer to be terminated by the complete cages (29 or 34 Å cages, respectively) as energetically stable terminations.

**Fig. 4:**
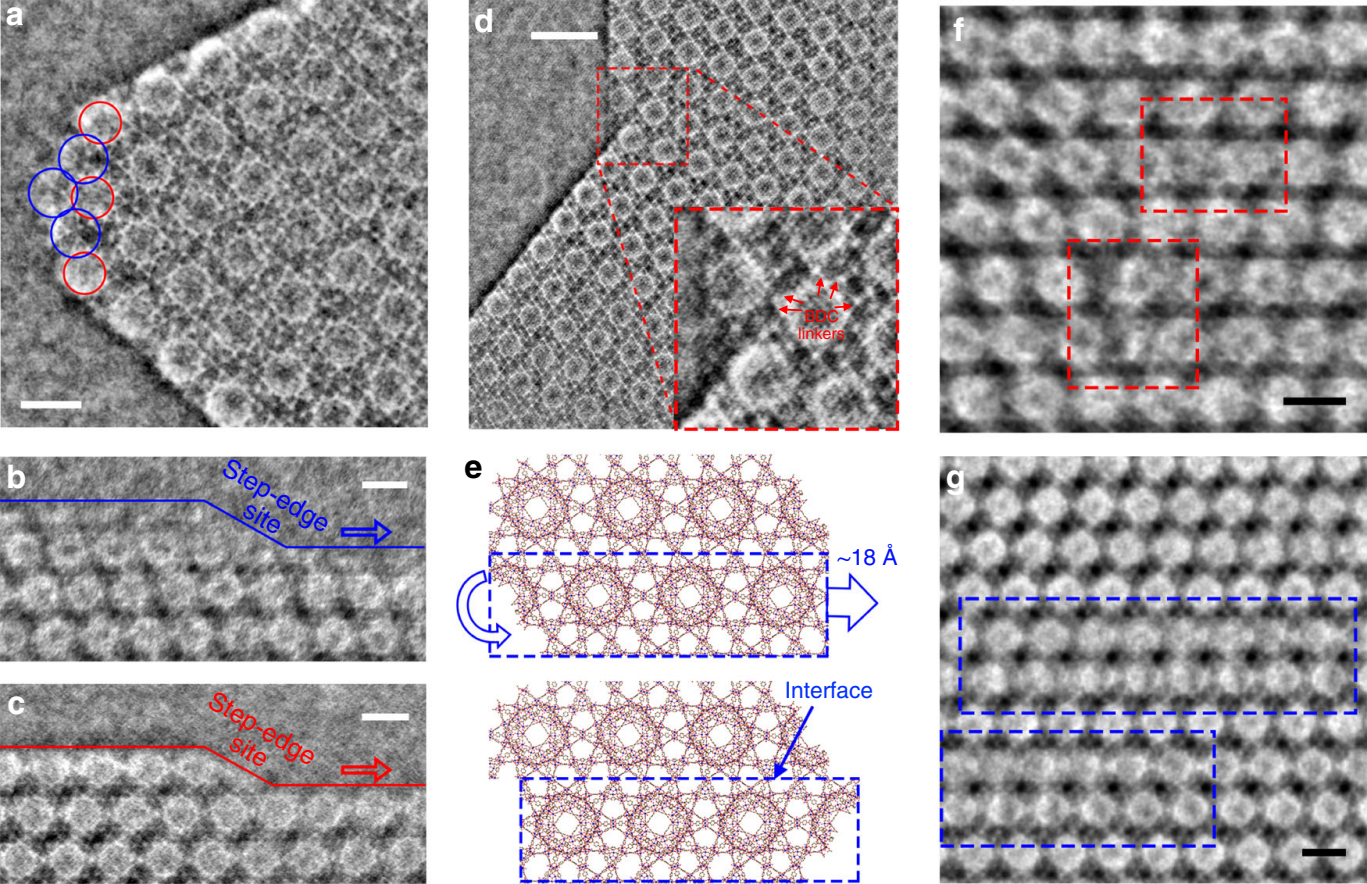
Revealing the local structures in the MIL-101 crystals. **a** The iDPC-STEM image showing the terminations of a corner in the octahedral MIL-101 crystal. The 34 Å cages (marked by the blue circles) were exposed at this corner. Scale bar, 5 nm. **b**, **c** The iDPC-STEM images showing the surface steps and step-edge sites of the MIL-101 crystals with two types of surface terminations, respectively. Scale bars, 5 nm. **d** The iDPC-STEM image showing the interface structures at a twin-plane boundary. Scale bar, 10 nm. **e** The structural model of the twin-plane interface imaged in **d**. The interface was formed by horizontally flipping and moving the marked part (below) to match the part above again. **f**, **g** The iDPC-STEM images showing the line (**f**) and plane (**g**) defects on the twin-plane interface. These defects lasted through the whole atom arrays parallel to the electron beam. Scale bars, 5 nm.

Preserving and imaging the surface structures and defects need more difficult efforts, as they are more sensitive to the electron beam than the crystalline bulk. The imaging capacity of low-dose iDPC-STEM at the local structures is quite comparable with those using the direct-detection electron-counting camera and cryogenic conditions^32–36^. Such high-resolution imaging of bulk lattices and surface terminations are helpful for us to understand the detailed local structures at the corners, edges, interfaces, and defects of the MIL-101 crystals. First, Fig. 4a revealed the structure of a corner in the octahedral MIL-101 crystal. At this corner, the imaged Cr/BDC super tetrahedrons indicated a surface termination with exposed 34 Å cages as marked by the blue circles in Fig. 4a, whereas the adjacent edges maintained the exposed 29 Å cages on the surfaces. Then, in Fig. 4b, c, we also observed the surface steps and step-edge sites with two types of terminations, and the growth of pores initiated at these step-edge sites and propagated along the {111} surfaces.

### Resolving the interface structures of MIL-101 twin crystals

Furthermore, we imaged the assembly of octahedral MIL-101 crystals and revealed the structures of twin-plane interfaces. Figure 4d and Supplementary Fig. 9 show the surface-to-surface assembly of two octahedral crystals viewed from the <110> projection, where the {111} surfaces were attached and connected without lattice mismatch. The connection of characteristic spheres on this interface can be further unraveled based on the direct imaging of super tetrahedrons between them. In addition, the schematic model of interface structure is given in Fig. 4e. The original lattices below were horizontally flipped and moved for a distance of ~18 Å, and then the two parts of lattices were bridged by the super tetrahedrons with little distortion to form the matched interface. Meanwhile, along these interfaces, there could be many dislocations due to the local defects of missing nodes and linkers (Supplementary Fig. 10). Figure 4f, g showed these defects on the interface, where partial nodes and linkers of MIL-101 were missing and the imaged characteristic spheres were imperfect. Such dislocated interfaces were generated from different sites at different surface steps. Thus, the mismatch between these interfaces will result in the missing of the partial nodes and linkers to maintain the ordered arrangement of the lattices around these defects. These results revealed the assembly of MOF nanocrystals, which is an interesting and general behavior proper to be studied by the real-space imaging methods. The high-resolution iDPC-STEM images allowed us observing the node-linker coordination at these local structures to better understand the synthesis and assembly of MOFs.

### The iDPC-STEM imaging of other beam-sensitive materials

**Fig. 5:**
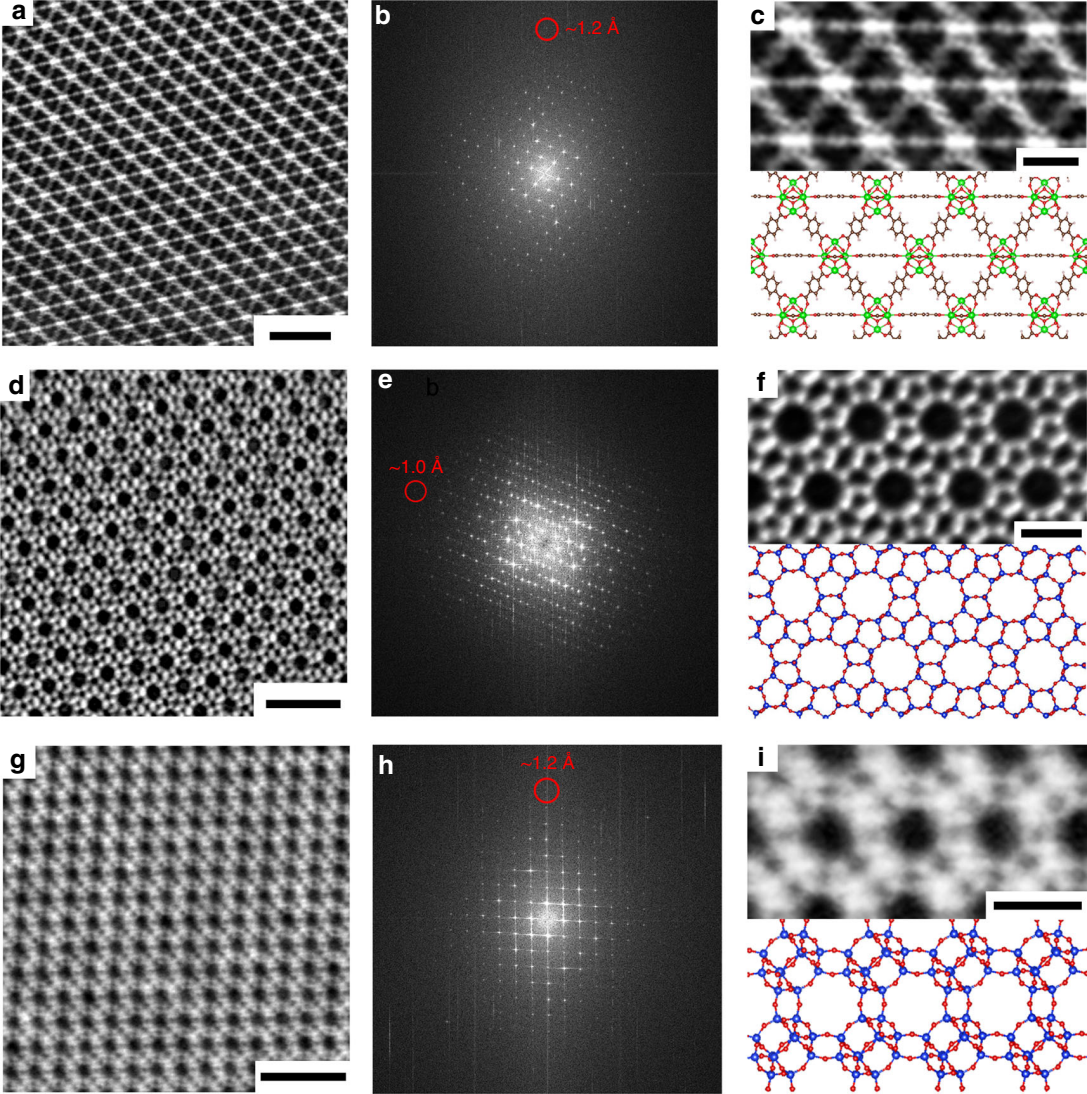
The iDPC-STEM imaging of other beam-sensitive materials. **a** The iDPC-STEM image of UiO-66 framework from the <110> projection. Scale bar, 3 nm. **b** The FFT pattern corresponding to **a** showing an information transfer of ~1.2 Å. **c** The magnified image of **a** compared with the structural model. Scale bar, 1 nm. **d** The iDPC-STEM image of ZSM-5 framework from the <010> projection. Scale bar, 3 nm. **e** The FFT pattern corresponding to **d** showing an information transfer of ~1 Å. **f** The magnified image of **d** compared with the structural model. Scale bar, 1 nm. **g** The iDPC-STEM image of SAPO-34 framework from the <1 -1 -1> projection. Scale bar, 3 nm. **h** The FFT pattern corresponding to **g** showing an information transfer of ~1.2 Å. **i** The magnified ima**g**e of **g** compared with the structural model. Scale bar, 1 nm.

Besides the MIL-101 framework, the iDPC technique can also be applied to investigate many other beam-sensitive frameworks, including another MOF, UiO-66, and zeolite frameworks (Supplementary Figs. 11–13). Figure 5a shows the iDPC-STEM images of UiO-66 crystals from the <110> projection. The information transfer in FFT pattern (Fig. 5b) reached to ~1.2 Å. In the magnified image compared with the structural model (Fig. 5c), we can clearly observe the node-linker coordination inside the framework and even the benzene ring structures can also be recognized. Both the heavy and light elements in the UiO-66 were identified with proper contrasts in one image, which confirmed the outstanding capacity of iDPC technique on the imaging of beam-sensitive materials and light-element species again. These results represent the highest resolution (~1.2 Å) of UiO-66 ever imaged by the electron microscopes together with a sufficient signal-to-noise ratio.

Then, two beam-sensitive Si/O-based zeolites, ZSM-5 and SAPO-34, were also imaged under the iDPC-STEM with ultrahigh resolution. The image in Fig. 5d shows the typical straight channels of ZSM-5 from the <010> projection. The corresponding FFT pattern in Fig. 5e indicated the resolution of ~1 Å for the ZSM-5. In the magnified image (Fig. 5f), the straight channels formed by Si_10_-rings can be atomically resolved, which are highly consistent with the structural model. Figure 5g exhibits the channel systems in the SAPO-34 framework with the information transfer of ~1.2 Å (FFT in Fig. 5h). We can observe the arranged channels formed by Si_8_-rings from the <1 -1 -1> projection when comparing the magnified image with the structural model in Fig. 5i. To the best of our knowledge, this is the first time we can obtained the atomic images of the SAPO-34 zeolite. These two kinds of zeolites have been widely used in the catalysis of various industrial applications. The atomic imaging of these catalyst structures can provide new insights on the mechanisms of diverse macroscopic processes at nanoscale, including the synthesis and catalysis.

In summary, with the assistance of the iDPC-STEM, we achieve the atomic-resolution imaging of MIL-101 crystals with visible giant cages and super tetrahedrons consisting of Cr nodes and BDC linkers. Based on these observations, we revealed two kinds of {111} surface terminations with different types of cages exposed respectively. Then, we also observed the structures and evolutions of surfaces, interfaces and defects in the self-assembled crystals. These local structures allowed us to better understand the node-linker coordination and the structure-property relations in MOFs. All these results proved the natural advantages of the iDPC technique for the low-dose and light-element imaging under the STEMs. The iDPC-STEM can also be used to resolve various other materials, which are sensitive to electrons and mainly composed of light elements, such as the zeolites, polymers, and 2D layered materials, and then provides the possibility to characterize these materials after their defects and surfaces are probed and modified.

## Methods

### Synthesis of MIL-101 crystals

MIL-101 was prepared by a hydrothermal method. Typically, 0.80 g of Cr(NO_3_)_3_⋅9H_2_O (2 mmol), 0.33 g of BDC (2 mmol), 2 mmol of HF (48 wt %), and 10 mL deionized water were successively added into a 100 mL stainless-steel autoclave lined with polytetrafluoroethylene. After heating at 220 °C for 8 h, the mixture was cooled down first to 150 °C in 1 h, and then slowly to room temperature in 12 h. Subsequently, the mixture was first filtrated by using a large pore fritted glass filter (2, Schott) to isolate large BDC crystals, and then filtrated in a small pore paper filter (1°, Whatman) to remove the free BDCs. The obtained green MIL-101 powder was soaked in ethanol (95% EtOH with 5% water) at 80 °C for 24 h followed by filtrating the hot solution. The solid was finally dried at 150 °C under vacuum for 24 h.

### The iDPC-STEM imaging

The high-resolution iDPC-STEM images were obtained under a Cs-corrected STEM (FEI Titan Cubed Themis G2 300) operated at 300 kV with a convergence semi-angle of 10 mrad. The STEM was equipped with a DCOR+ spherical aberration corrector for the electron probe, which was aligned using a standard gold sample before observations. The aberration coefficients we used were shown as following: C1 = 1.53 nm, A1 = 2.22 nm, A2 = 10.7 nm, B2 = 22.5 nm, C3 = 996 nm, A3 = 794 nm, S3 = 282 nm, A4 = 4.99 µm, D4 = 2.5 µm, B4 = 6.38 µm, C5 = −954 µm, A5 = 201 µm, S5 = 338 µm, and R5 = 42.1 µm. Four images used for 2D integration were acquired by a four-quadrant DF4 detector with an optional high-pass filter applied to reduce the low-frequency information in the image. The electron-beam current was reduced lower than 0.1 pA, which was measured by the Faraday cup. The collection angles of iDPC-STEM were set as 4 ~ 20 mrad. Then, the dwell time of Fig. 2e is 16 μs and the pixel size of Fig. 2e is 0.5027 Å. Thus, the electron dose was calculated using the equation: (beam current) × (dwell time)/(pixel size)^2^ < 40 *e*^−^/Å^2^. The projected potential was simulated by the QSTEM software based on the multislice method. The parameters used in simulation were consistent with those in the experiments.

### Other characterizations

The PXRD results of MIL-101 crystals were obtained using a diffractometer (Rigaku D/Max-RB) with the CuKα radiation at 40 kV and 120 mA. The SEM images were obtained under a high-resolution SEM (JEOL, JSM-7401) at 1 kV. The ADF-STEM images were also obtained under the Cs-corrected STEM (FEI Titan Cubed Themis G2 300) operated at 300 kV with the same convergence semi-angle, collection angle and aberration coefficients as those in iDPC-STEM imaging. The electron-beam current of ADF-STEM was set as 0.7 ~ 3.2 pA.

## Supplementary information

Supplementary Information

## Data Availability

The authors declare that all relevant data supporting the findings of this study are available within the paper and its Supplementary Information files. Additional data are available from the corresponding authors upon reasonable request.
