# Supplementary material for: Imaging the node-linker coordination in the bulk and local structures of metal-organic frameworks
Source: Nat Commun. 2020 Jun 1;11:2692. doi: 10.1038/s41467-020-16531-y (PMC7264187; doi:10.1038/s41467-020-16531-y)
Supplement: Supplementary file 1 — Supplementary Information [file 41467_2020_16531_MOESM1_ESM.pdf]

Supplementary Information

Supplementary Figures

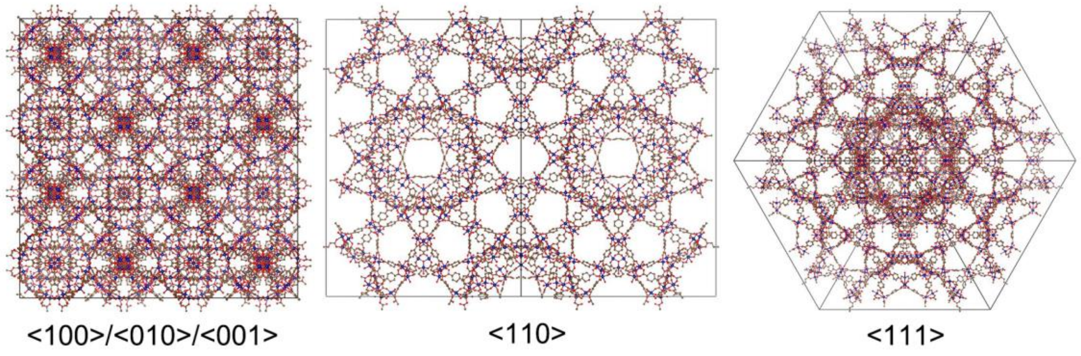

Supplementary Figure 1 | The structural model of MIL-101 framework.

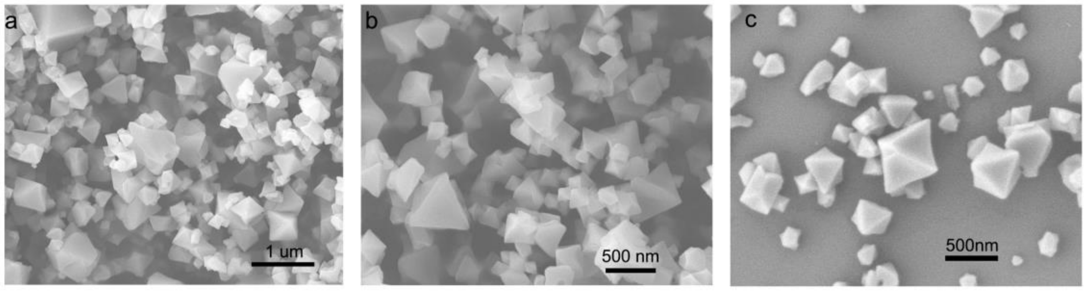

Supplementary Figure 2 | The SEM images of MIL-101 powder.

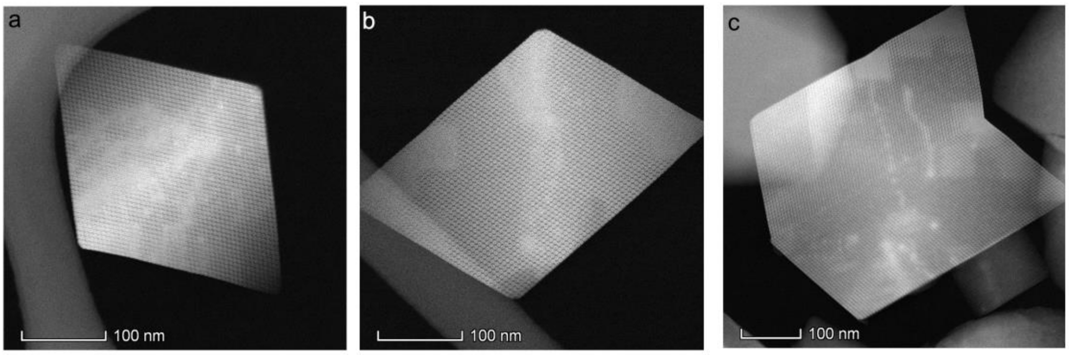

Supplementary Figure 3 | The ADF-STEM images of individual MIL-101 crystals with different morphologies.

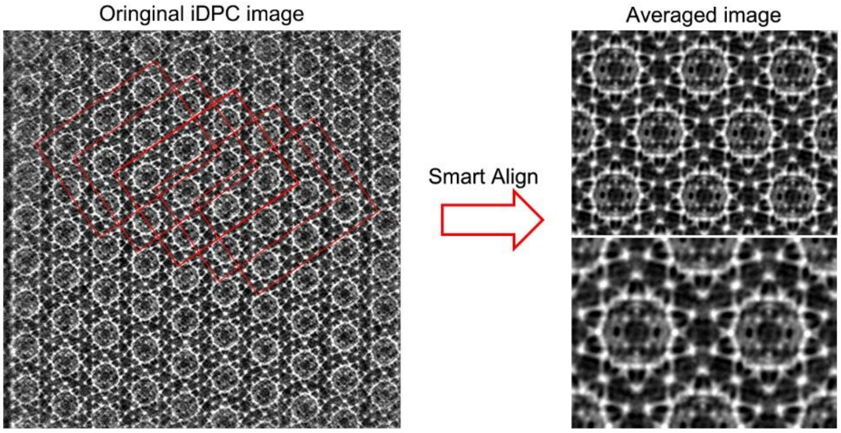

Supplementary Figure 4 | In the iDPC-STEM image, the periodically-selected parts are marked by the red frames. Here we only show 6 red frames, while, totally, over 30 images were used to obtain the averaged image after the stacking process. Using the Smart Align software, these selected parts were stacked to further enhance the signal-to-noise ratio and confirm the detailed structures in the MIL-101 frameworks.

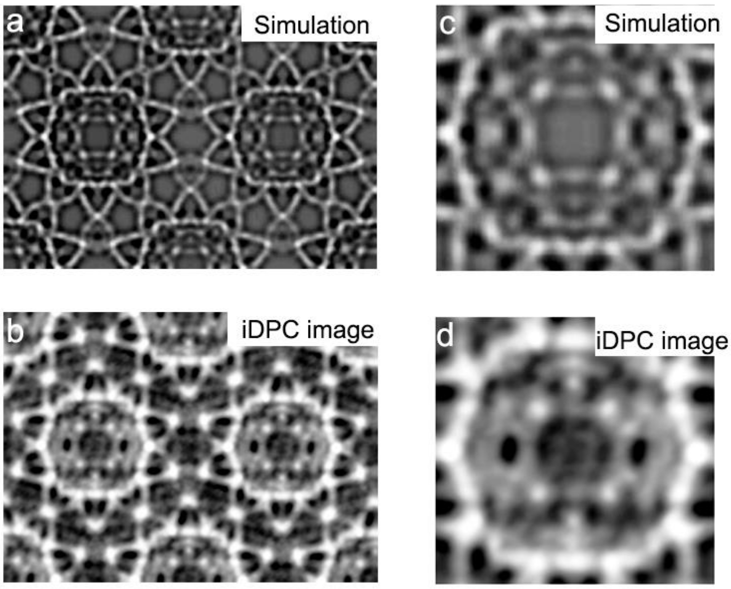

Supplementary Figure 5 | The comparison between the iDPC-STEM images and simulation results. The projected potential was simulated by the QSTEM software based on the multislice method with the point spread function width of 1.8 Å.

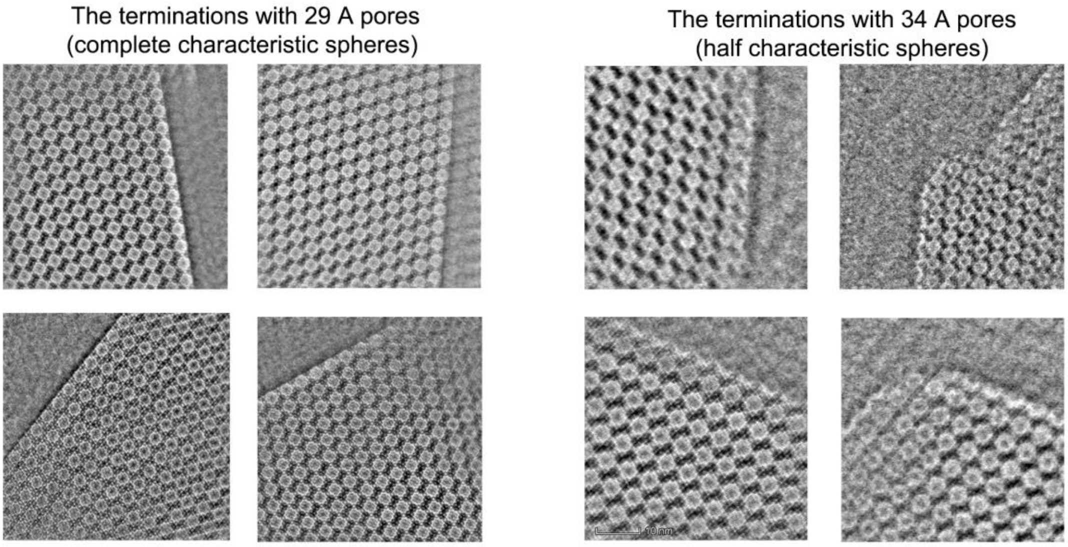

Supplementary Figure 6 | More iDPC-STEM images showing two types of surface terminations respectively.

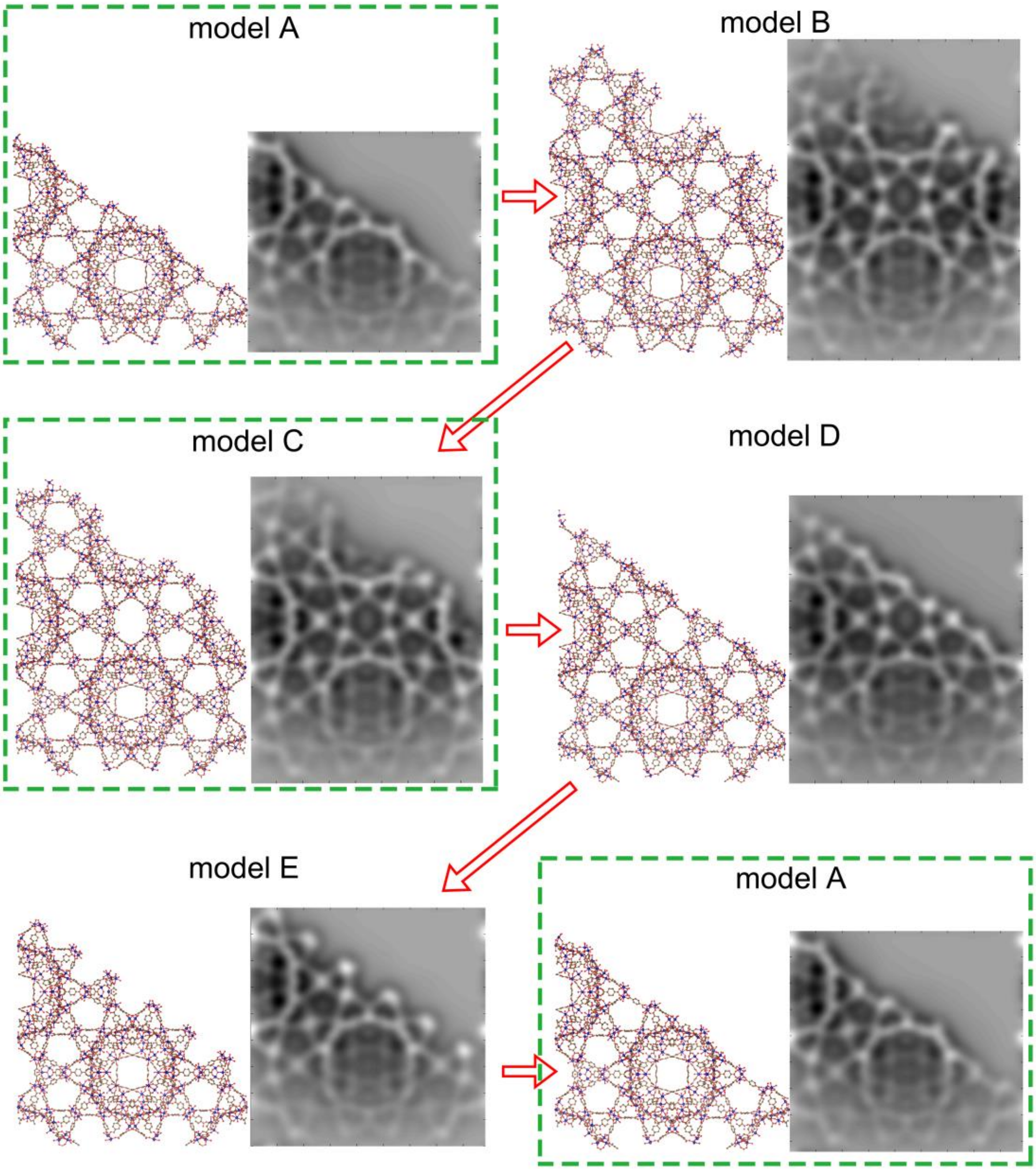

Supplementary Figure 7 | Five possible surface models and the corresponding simulated potentials (with resolution of 5 Å). The state A and C are consistent with the observed two types of surfaces by iDPC-STEM.

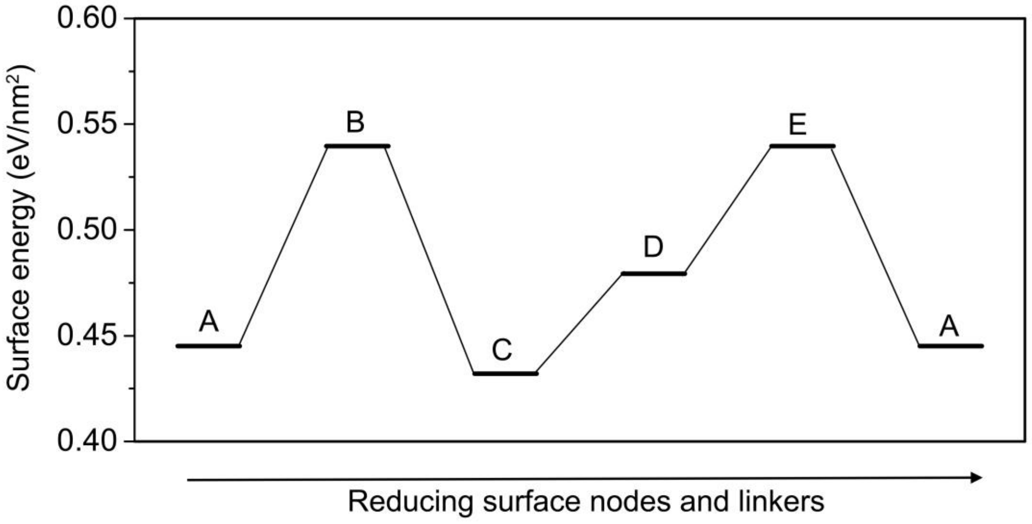

Supplementary Figure 8 | The calculated energies of five surface states in Supplementary Figure 7.

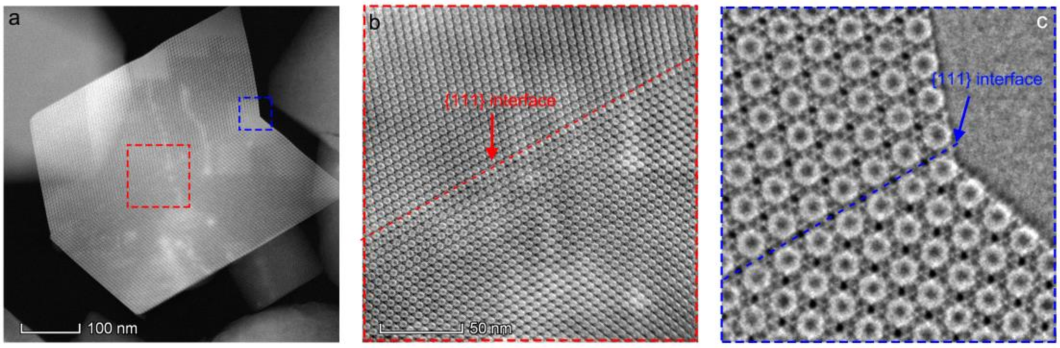

Supplementary Figure 9 | (a) The ADF-STEM image of a twin-crystal of MIL-101. (b) The ADF-STEM image of the {111} interface marked as the red frame in a. (c) The iDPC-STEM image of the {111} interface marked as the blue frame in a.

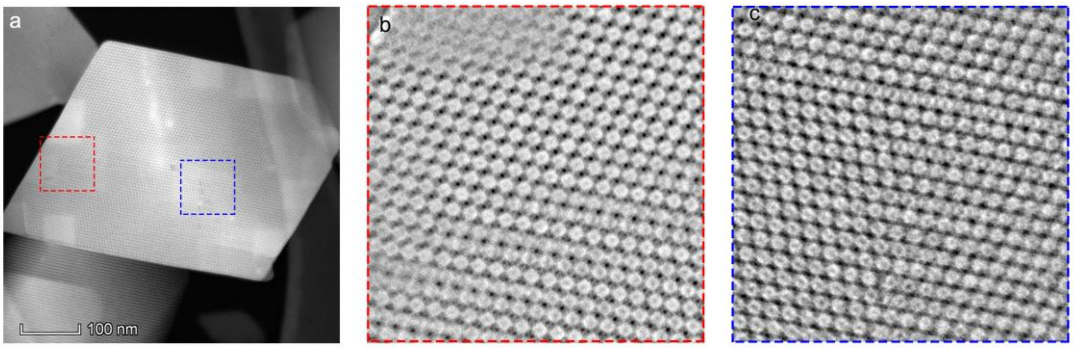

Supplementary Figure 10 | (a) The ADF-STEM image of a MIL-101 crystal. (b and c) The iDPC-STEM images showing the defects in this MIL-101 crystal.

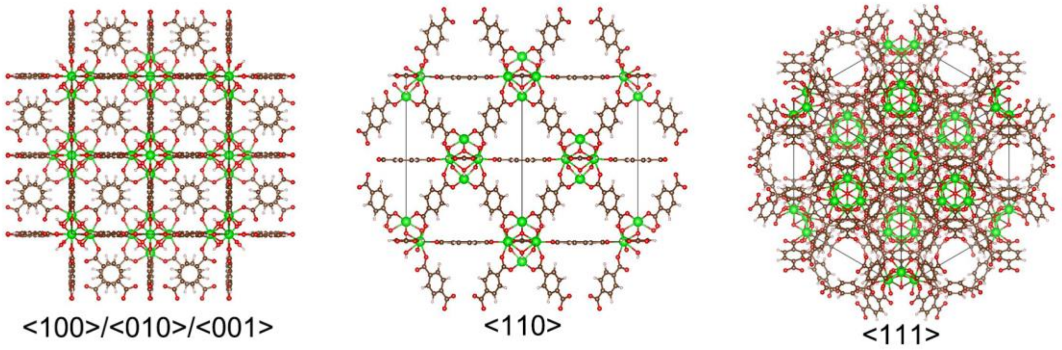

Supplementary Figure 11 | The structural model of UiO-66 framework.

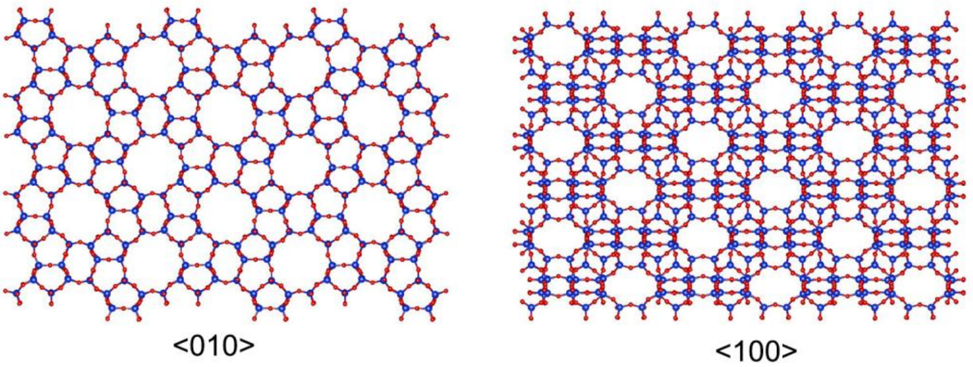

Supplementary Figure 12 | The structural model of ZSM-5 framework.

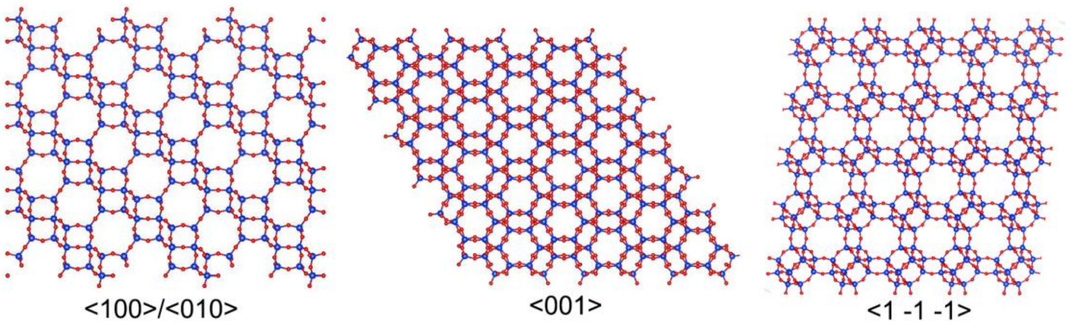

Supplementary Figure 13 | The structural model of SAPO-34 framework.

Supplementary Discussion

In order to identify the surface structure and energy, we established five possible models of the {111} surfaces in single units by gradually reducing the surface nodes and linkers layer by layer from the [111] direction. These five models are numbered from A to E in Supplementary Fig. 7. During the modelling, we followed these principles: try to keep the surface flat; try to keep the highest coordination number of surface nodes (reduce the dangling bonds); do not consider the uncoordinated surface ligands. And then, we obtained the simulated potentials of the single units in five models in Supplementary Fig. 7. The point spread function width (resolution) was set to 5 Å. After comparing

these simulated results with the two types of surfaces we imaged in Fig. 3, it is obvious that the surface terminations we observed by iDPC-STEM are consistent with the models A and C respectively.

We calculated the surface energy of five models and analyzed the surface stability. First, we calculated the energy of single Cr node ($E_{Cr(N)}$) with different coordination number N. The calculations were performed using the Vienna Ab initio Simulation Package (VASP 5.4.5)[1]. The effect of the core electrons in the valence density was taken into account by means of the projected augmented wave (PAW) formalism[2], and the exchange-correlation functional is the Perdew-Burke-Ernzerhof (PBE) functional[3]. The valence density was expanded in a plane wave basis set with a kinetic energy cutoff of 400 eV. The energy convergence criteria are 1.0×10^{-5} eV. Integration in the reciprocal space was carried out at the Γ k-point of the Brillouin zone. Then, the dangling bond energy $E_{d(N)}$ was calculated by the equation: $E_{d(N)} = E_{Cr(N)} + (6-N) \times E_{BDC} - E_{Cr(N=6)}$, where E_{BDC} is the energy of single free BDC molecule. The results are $E_{d(N=6)}$= 0 eV, $E_{d(N=5)}$= 1.14 eV, $E_{d(N=4)}$= 1.83 eV, $E_{d(N=3)}$=3.71 eV. When $N<3$, the energy is too high, and such nodes don't exist in our surface models. In the calculation of total surface energy, we didn't consider the structural changes of surface nodes, and attributed the total surface energy mainly to the dangling bond energy of uncoordinated Cr nodes.

Then, for the five surface models in Supplementary Fig. 7, we counted the numbers of surface nodes with different coordination numbers within a same surface area of 11.0×12.5 nm^2. And we summed their dangling bond energies as the total surface energy within this surface area, and obtained the surface energies per nm^2 of five surface models as shown in Supplementary Fig. 8. According to the previous results, under the synthesis conditions (with HF and 150 °C heating in vacuum), the surface BDC ligands will gradually detach from the nodes[4]. In Supplementary Fig. 8, from left to right, we provided a cycle of surface states (A to E) where the nodes and BDC linkers (ligands) were released layer by layer. And the state A and C corresponding to the two observed surfaces in Fig. 3 show the lower surface energies (as energy valleys). As we mentioned in the main text, the surfaces at state A and C were terminated by complete 29 and 34 Å cages respectively. When we further opened these cages on the surfaces, the coordination number of surface nodes decreased, and the surface energy increased in the state B, D and E. Thus, it is necessary to overcome a larger energy barrier to release the surface nodes and ligands from the state A and C. In summary, the state A and C are the most possible surfaces under this synthesis condition, and they show the similar energies based on thermodynamics. In a plenty of statistics and observations, we found these two surface states co-existed in the same specimen and there were almost only these two states. These calculations perfectly explained our observations on the surface structures of MIL-101 crystals.

Supplementary References

1. Kresse, G., Furthmüller, J. Efficient iterative schemes for ab initio total-energy calculations using a plane-wave basis set. Phys. Rev. B: Condens. Matter Mater. Phys. 54, 11169-11186 (1996).

2. Blöchl, P. E. Projector Augmented Wave Method. Phys. Rev. B: Condens. Matter

Mater. Phys. 50, 17953-17979 (1994).
3. Perdew, J. P. et al. Atoms, Molecules, Solids, and Surfaces: Applications of the Generalized Gradient Approximation for Exchange and Correlation. Phys. Rev. B: Condens. Matter Mater. Phys. 46, 6671-6687 (1992).
4. Li, X. et al. Direct imaging of tunable crystal surface structures of MOF MIL-101 using high-resolution electron microscopy. J. Am. Chem. Soc. 141, 12021-12028 (2019).
